# Deep Learning Multi-label Tongue Image Analysis and Its Application in a Population Undergoing Routine Medical Checkup

**DOI:** 10.1155/2022/3384209

**Published:** 2022-09-29

**Authors:** Tao Jiang, Zhou Lu, Xiaojuan Hu, Lingzhi Zeng, Xuxiang Ma, Jingbin Huang, Ji Cui, Liping Tu, Changle Zhou, Xinghua Yao, Jiatuo Xu

**Affiliations:** ^1^Basic Medical College, Shanghai University of Traditional Chinese Medicine, 1200 Cailun Road, Shanghai 201203, China; ^2^Department of Acupuncture and Moxibustion, Huadong Hospital, Fudan University, 221 West Yanan Road, Shanghai 200040, China; ^3^Shanghai Collaborative Innovation Center of Health Service in Traditional Chinese Medicine, Shanghai University of Traditional Chinese Medicine, 1200 Cailun Road, Shanghai 201203, China; ^4^School of Information Science and Engineering, Xiamen University, Xiamen 361005, China

## Abstract

**Background:**

Research on intelligent tongue diagnosis is a main direction in the modernization of tongue diagnosis technology. Identification of tongue shape and texture features is a difficult task for tongue diagnosis in traditional Chinese medicine (TCM). This study aimed to explore the application of deep learning techniques in tongue image analyses.

**Methods:**

A total of 8676 tongue images were annotated by clinical experts, into seven categories, including the fissured tongue, tooth-marked tongue, stasis tongue, spotted tongue, greasy coating, peeled coating, and rotten coating. Based on the labeled tongue images, the deep learning model faster region-based convolutional neural networks (Faster R-CNN) was utilized to classify tongue images. Four performance indices, i.e., accuracy, recall, precision, and F1-score, were selected to evaluate the model. Also, we applied it to analyze tongue image features of 3601 medical checkup participants in order to explore gender and age factors and the correlations among tongue features in diseases through complex networks.

**Results:**

The average accuracy, recall, precision, and F1-score of our model achieved 90.67%, 91.25%, 99.28%, and 95.00%, respectively. Over the tongue images from the medical checkup population, the model Faster R-CNN detected 41.49% fissured tongue images, 37.16% tooth-marked tongue images, 29.66% greasy coating images, 18.66% spotted tongue images, 9.97% stasis tongue images, 3.97% peeled coating images, and 1.22% rotten coating images. There were significant differences in the incidence of the fissured tongue, tooth-marked tongue, spotted tongue, and greasy coating among age and gender. Complex networks revealed that fissured tongue and tooth-marked were closely related to hypertension, dyslipidemia, overweight and nonalcoholic fatty liver disease (NAFLD), and a greasy coating tongue was associated with hypertension and overweight.

**Conclusion:**

The model Faster R-CNN shows good performance in the tongue image classification. And we have preliminarily revealed the relationship between tongue features and gender, age, and metabolic diseases in a medical checkup population.

## 1. Introduction

Tongue inspection is the most common, intuitive, and effective diagnostic method of traditional Chinese medicine (TCM) [1]. Recent TCM researches have realized measurable and digitized color features of tongue images by means of color space parameters such as RGB, Lab, and HIS [2–4].

However, the quantification of the shape and texture of tongue images remains a difficult point in tongue diagnosis. Much attention has focused on the automatic recognition methods of the shape and texture of tongue images. Obafemi-Ajayi et al. [5] have proposed a feature extraction method for automated tongue image shape classification based on geometric features and polynomial equations. Yang et al. [6] extracted the cracks by applying the G component of the false-color image in RGB color space, and the accuracy of detection was 82.00%. Douglas–Peucker algorithm was implemented to extract the number of features for tooth-marked tongue and achieved an accuracy of 80% [7]. Xu et al. [8] conducted an RGB color range and a gray mean value of acantha and ecchymosis in tongue patterns, and the overall accuracy of recognition was 77.10%. Wang et al. [9] realized the prickles extraction on the green tongue image, with an accuracy of 88.47%. Yet, due to the complex and diverse tongue features, classical image processing methods have some problems, such as space-time consumptive algorithm, difficulties in automated high-throughput processing, and weak migration ability in correlation research [10–12], which make the comprehensive analysis of tongue images unavailable.

Intelligent diagnosis based on images is a main direction of modernization of tongue diagnosis technology [13]. As the current mainstream technology, a convolutional neural network (CNN) has a powerful capability of feature extraction and representation [14, 15], which greatly improves the accuracy and efficiency of tongue image segmentation, and classification [16–20]. For example, Chen's team has utilized the deep residual neural network (ResNet) to identify the tooth-marked tongue, with an accuracy of over 90% [21]. Xu et al. [22] have proposed a CNN model combining a u-shaped net (U-NET) and Discriminative Filter Learning (DFL) for classification and recognition of different types of tongue coating, achieving an F1-Score of 93%. The research on the recognition and classification of the tooth-marked tongue [23] and cracked tongue [24] has significantly improved the accuracy of tongue image identification.

**Figure 1:**
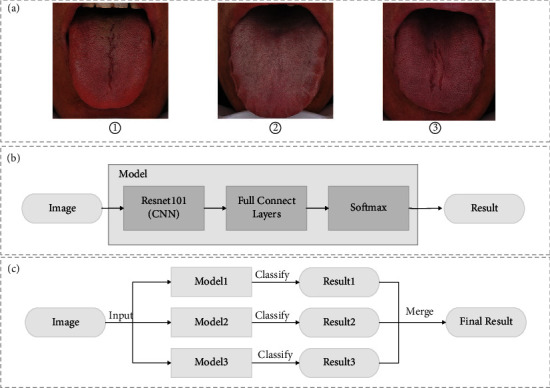
Deep learning methods for tongue diagnosis analyses. (a) Fissured tongue image, tooth-marked tongue image, and tongue image with fissures and tooth marks; (b) CNN method of single-object detection; (c) CNN method of multi-label detection in tongue images.

However, tongue images have multi-label attributes (Figure 1(a)). Although the classical model CNN shows better recognition performance for single tongue features such as tooth marks or fissures (Figure 1(b)), the multi-CNN fusion model has no apparent superiority in the multi-label classification of tongue images with diverse features (Figure 1(c)). Under nonparallel conditions, multiple CNN models require huge space and time. The classical CNN model fails to accurately identify, locate and quantify complex and diverse fine-grained features of tongue images simultaneously, and it is difficult to achieve efficient detection and recognition of tongue images in parallel.

Object detection technology is considered as a method to find a specific object in an image and determine the specific position of the object. As one of the mainstream neural networks for object detection, faster region-based convolutional neural networks (Faster R-CNN) [25] can perform multi-label recognition with only one model, thus reducing the cost of training multiple models. Here, we utilized Faster R-CNN and fine-tune method to extract local features of tongue images, learning the high-level semantic features. Aiming at 7 categories of tongue shape and texture in TCM, ResNet [26] was used as the backbone network for feature extraction to construct a deep learning model.

In this research, we constructed a standard database for training, testing, and validation realized the efficient and accurate classification and recognition of local features of tongue images and applied it to a population undergoing medical checkups with Chinese medicine, in order to reveal the association of tongue image features with diseases.

## 2. Materials and Methods

**Figure 2:**
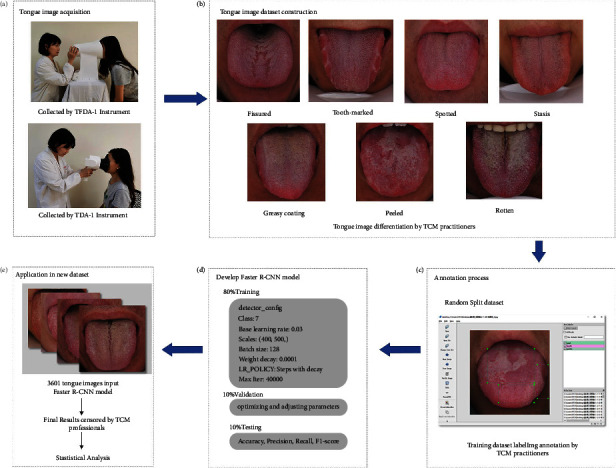
The workflow of the entire research. (a) Demonstration of the acquisition process of tongue image; (b) example samples of tongue image differentiation dataset calibrated by experts; (c) interest regions marked manually by TCM practitioners using the LabelImg software; (d) Faster R-CNN model trained by tongue images and object location of training set; (e) study on tongue image features of 3601 people undergoing medical checkup based on Faster R-CNN model.

We proposed a deep learning multi-label tongue image model based on Faster R-CNN. A total of 8676 tongue images were collected to train and test the proposed model. The collected tongue images annotated by experts were divided into seven categories. Furthermore, this approach was applied to a population undergoing medical checkups with Chinese medicine. The specific process of this study is shown in Figure 2.

### 2.1. Tongue Image Collection and Preprocess

**Figure 3:**
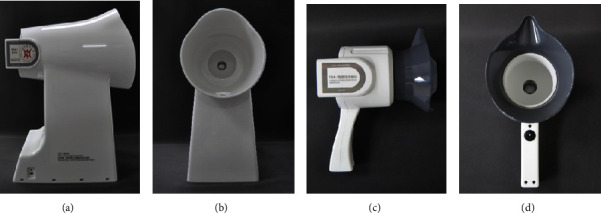
TFDA-1 and TDA-1 tongue diagnosis instrument. (a) Side view of TFDA-1; (b) front view of TFDA-1; (c) side view of TDA-1; (d) front view of TDA-1.

As shown in Figure 3, all the tongue images were acquired by using TFDA-1 and TDA-1 tongue diagnosis instruments designed by Xu's team at Shanghai University of TCM. The instruments were equipped with unified CCD equipment, a standard D50 light source, a color temperature of 5003K, and a color rendering index of 97 [27]. Tongue images were obtained from September 2017 to December 2018 at Shuguang Hospital. The raw tongue image size was 5568 × 3711 pixels in JPG format. To reduce the amount of deep learning calculation and eliminate the interference of other regions except the tongue body, all tongue images were automatically cut to the size of 400 × 400 pixels by mask R-CNN.

### 2.2. Tongue Image Labeling and Datasets Construction

All tongue image labels were evaluated and screened by 10 TCM experts with normal vision and reported normal color vision [28]. To avoid the chromatic differences from the monitor, experts interpreted and screened under uniform conditions with 27 inches APPLE Cinema HD monitor. With reference to the diagnostic criteria of tongue image features [1, 29], tongue images were divided into seven categories. At least 8 out of 10 experts confirmed that the dataset contained the same labels, and all 8676 tongue images were annotated by two experts as seven different folders. Example samples of each typical tongue image were shown in Figure 2(b), and the other eight experts respectively checked the labeled folders. The images with the inconsistent diagnosis were excluded from this research.

The datasets for Faster R-CNN were with the MS COCO format, which was the most popular standard format in the field of object detection [30]. We used LabelImg (Version 1.8.1) to annotate the interest regions of shape and texture on the tongue image. The annotation was confirmed by experts and the process interface is shown in Figure 2(c). Then, the generated “XML” annotation files were transferred into the “JSON” format file using Python (Version 3.6).

### 2.3. Dataset Partition

The constructed datasets were randomly partitioned into 80% training set, 10% validation set, and 10% testing set. The number of the training datasets and labels in 7 categories used to build the Faster R-CNN model were shown in Table 1. In addition, the number of tongue images in each category included in the testing set was equal to the validation set.

### 2.4. Faster R-CNN Model Development for Recognizing Tongue Shape and Texture

**Figure 4:**
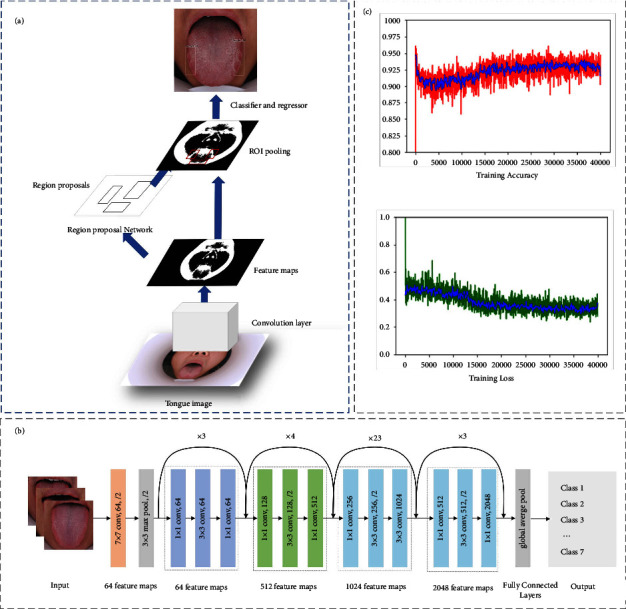
Faster R-CNN Model development for recognizing tongue shape and texture. (a) The workflow of Faster R-CNN; (b) the architecture of backbone feature extraction network ResNet101; (c) the accuracy and loss of model training.

Figure 4(a) shows the network architecture diagram of Faster R-CNN, mainly consisting of four parts: convolution layer, regional proposal network, the region of interest (ROI) pooling layer, and a layer of classifier and regressor [31]. The backbone convolution layer ResNet101, as shown in Figure 4(b), extracts feature maps from the input tongue images; the region proposal network (RPN) centers on each pixel of the feature maps, and generated anchor boxes with different scales in the tongue images by using nonmaximum suppression; the ROI pooling computes feature maps for region proposals; the final output feature maps of the ROI pooling layer are performed for classification. Finally, an average pooling is applied, and the features obtained from the pooling are used for classification and bounding box regression, respectively.

### 2.5. Model Training, Validation, and Testing

The Faster R-CNN model based on the Caffe framework was deployed in the Ubuntu operating system by using open-source code and was trained in a computing environment with 4 NVIDIA GTX 1080Ti GPUs, 12 Intel Core I7-6850K CPUs, and 128 GB DDR4 RAM.

The model training, validation, and testing were conducted according to the following steps: First, the Faster R-CNN network was fine-tuned on a tongue image dataset for 40000 iterations with an optimizer of stochastic gradient descent (SGD), the learning rate of 0.03, weight decay of 0.0001, the momentum of 0.9, gamma value of 0.1, and batch size of 128. Detailed initial parameters were shown in Table 2.

Then, the tongue image and the marked position information were fed into an integrated Faster R-CNN model for training. In each training iteration, features were extracted, labels and frame position were predicted, and losses (i.e., errors) between predicted frame position, predicted labels, and object actual position and object actual label were calculated. The parameters were updated according to the backward error propagation. Complete the training and generate the object detection model of tongue image of TCM. At the end of the training, a well-trained object detection model for tongue images was achieved. Collect and observe results using a well-trained model with different hyper-parameters over the validation set. The operation of validation was performed during the training process. Based on the results over the validation set, the state of the model was checked, and the hyperparameters of the model were adjusted. When the results of accuracy in the validation do not increase, the training is stopped. The loss function for Faster R-CNN sums the classification loss and regression loss, as defined in the following equation [24]:

(1) $$\begin{array}{c} L\left(p_{i},t_{i}\right)=\frac{1}{N_{\text{cls}}}\sum_{i}L_{\text{cls}}\left(p_{i},p_{i}^{*}\right)+\lambda \frac{1}{N_{\text{reg}}}\sum_{i}p_{i}^{*}L_{\text{reg}}\left(t_{i},t_{i}^{*}\right), \end{array}$$

where *N*_cls_ and *N*_reg_ are the number of anchors in minibatch and number of anchor locations, *λ* and *i* mean the selected anchor box index and the balancing parameter; *p*_*i*_ and *p*_*i*_^*∗*^  represent the predicted probability and the ground truth of tongue feature; *t*_*i*_ and *t*_*i*_^*∗*^ represent the predicted bounding box and actual tongue feature label box. The accuracy results and the loss changes in the training are depicted in Figure 4(b).

Finally, by adjusting the initial learning rate and comprehensive comparison, the training model with a learning rate of 0.001 and iteration of 40000 was finally selected as the final object detection model. Then the trained model was applied to the testing set.

### 2.6. Strategies for the Prevention of Overfitting

In this study, the two means of regularization and dropout were deployed to prevent overfitting. In the process of the training model, the regularization of L2 was leveraged to constrain the weight estimates, so as to help in preventing overfitting [32]. In addition, the technique of dropout was applied for training the last several classification layers in the neural network of Faster R-CNN. By means of the dropout, convolution kernels were randomly deactivated in the training process [33]. Furthermore, the importance of convolution kernels in the classification layers was dynamically balanced. Also, the overfitting phenomenon could be alleviated.

### 2.7. Indices for Model Evaluation

Based on the classification results of the model Faster R-CNN over the testing set, the five indices, including accuracy ((2)), recall ((3)), precision ((4)), and F1-score ((5)), were selected as metrics to evaluate the performance of Faster R-CNN in the multiclass classification of tongue images [34–37]. True positive (TP) means that the expert's conclusion and the result of object detection are the same, and false negative (FN) represents that the existing tongue feature category is not detected. False positive (FP) means if the tongue feature detection algorithm classifies those that are not in this category. True negative (TN) denotes that if the tongue image does not belong to a certain category, the tongue feature detection algorithm is the same as the expert conclusion. Macro averaged measures for the above indices are calculated for the model Faster R-CNN with respect to the 7-classes classification of tongue images.

(2) $$\begin{array}{c} \text{Precision}=\frac{\text{TP}}{\text{TP}+\text{FP}}, \end{array}$$

(3) $$\begin{array}{c} \text{Recall}=\frac{\text{TP}}{\text{TP}+\text{FN}}, \end{array}$$

(4) $$\begin{array}{c} \text{Accuracy}=\frac{\text{TP}+\text{TN}}{\text{TP}+\text{FP}+\text{TN}+\text{FN}}, \end{array}$$

(5) $$\begin{array}{c} F1-\text{score }=\frac{2\times \text{Precision}\times \text{Recall}}{\text{Precision }+\text{ Recall}}. \end{array}$$

### 2.8. Application of Tongue Image Detection Model

The Faster R-CNN model obtained from the above training was applied to the population undergoing routine medical checkups with Chinese medicine to explore the association between tongue features and diseases. All samples were collected from January 2019 to December 2019. A total of 3601 subjects were included in the physical examination center of Eastern Hospital of Shuguang Hospital affiliated with Shanghai University of TCM. We excluded women who were pregnant or nursing; those who cannot cooperate with researchers. All volunteers signed informed consent, all subjects completed routine medical checkups and simultaneously used the TFDA-1 tongue diagnosis instrument to capture tongue images.

All tongue images were analyzed by a trained Faster R-CNN model. All analysis and test results were verified by experts for the second time, and the analysis results were unanimously confirmed. If they were inconsistent, comprehensive analysis results should be adopted. The indicators of shape and texture features of tongue images were classified into two categories. Doctors at the physical examination center of Shuguang Hospital affiliated with Shanghai University of TCM made a diagnosis with reference to the corresponding clinical guidelines for diseases, aiming at the common and multiple diseases in the medical checkup population.

### 2.9. Statistical Analysis Methods

Excel and Python3.6 were used for data matching, merging, and sorting. The tongue images were described by percentage (%) and were compared using the Pearson *χ*^2^ test. Statistical analysis was performed using the IBM SPSS Statistics for Windows, version 25 (IBM Corp., Armonk, N.Y., USA). All results were compared using the two-tailed*t* test and differences were considered statistically significant when *P* < 0.05.

A complex network by the improved node contraction method [38–40] is a weighted network that contains the degree and weight of edges based on the obtained node importance. The weight of the weighted network was defined as

(6) $$\begin{array}{c} \partial \left(WG\right)=\frac{1}{s\times l}=\frac{1}{\sum_{i}^{n}N_{i}\sum_{j\in N_{i}}W_{ij}\times \sum_{i\neq j\in V}d_{ij}/n\times \left(n-1\right)}, \end{array}$$

The visualization tool Python NetworkX [41] was used to store the constructed network in the form of the adjacency matrix and triple, and the complex network diagram was built with disease and tongue image features as nodes.

## 3. Results

### 3.1. Tongue Image Detection Over Testing Set

In our testing set, the average accuracy of the model achieved 90.67%, with a precision of 99.28%, recall of 91.27%, and F1 score of 95.00%, indicating that the model had a good detection effect and can accomplish the multiobject recognition task well, as shown in Table 3.

**Figure 5:**
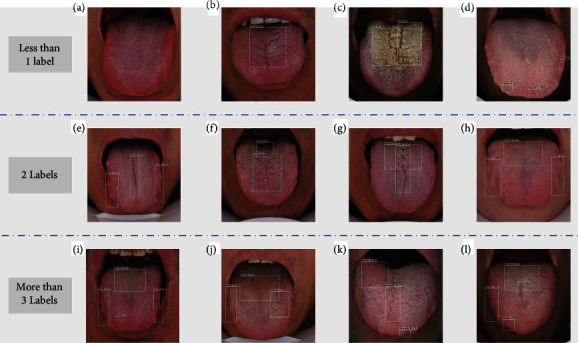
Examples of tongue image feature detection.

Our method detected tongue shape and texture features with different scales and ratios.  Figure 5(a) shows a normal tongue image (without tooth marks, fissures, stasis, spots, greasy coating, peeled coating, and rotten coating), and there was no mark in the test result; Figures 5(b)–5(d)) show tongue images with a single feature, (b) is a fissured tongue, (c) is a greasy coating (there are 2 spots), and (d) is a stasis tongue (there were 3 spots). Figures 5(e)–5(h)) show a combination of two different tongue images, in which (e) is a tooth-marked tongue with fissures, (f) is a peeled tongue with fissures, (g) is a greasy tongue with fissures, and (h) is a greasy tongue with tooth marks. Figures 5(i)–5(l)) show three or more different tongue-shaped features, in which (i) shows a greasy coating, tooth marks and stasis were detected simultaneously, (j) shows a greasy coating, tooth marks and spots were detected simultaneously, (k) shows a peeled coating, fissures and stasis were detected simultaneously, and (l) shows a greasy coating, tooth marks, fissures, and stasis were detected simultaneously.

### 3.2. Distribution of Tongue Image Features in the Medical Checkup Population

**Figure 6:**
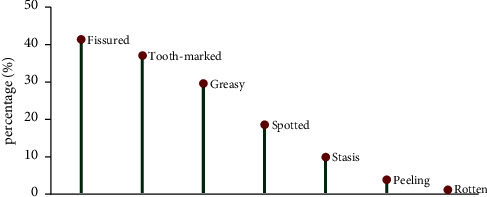
Distribution of different tongue shape and texture features.

The tongue images were input into the established optimal Faster R-CNN intelligent tongue diagnosis analysis model, and 1494 cases (41.49%) of the fissured tongue, 1338 cases (37.16%) of the tooth-marked tongue, 1068 cases (29.66%) of greasy coating, 672 cases (18.66%) of the spotted tongue, 359 cases (9.97%) of stasis tongue, 143 cases (3.97%) of peeled coating, and 44 cases (1.22%) of rotten coating, as shown in Figure 6.

### 3.3. Statistics of Tongue Image Features on the Gender Factors and the Age Factors

**Figure 7:**
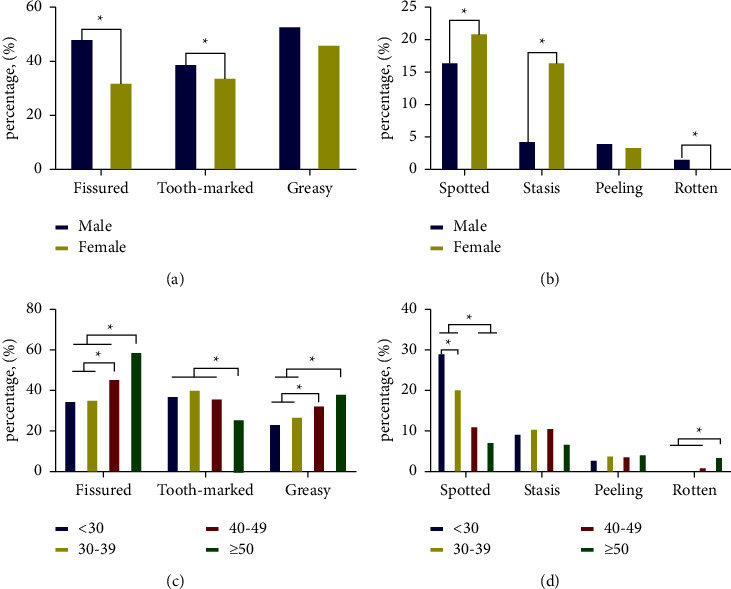
Comparison of tongue shape and texture features of different age ranges and genders.

It showed that the proportion of fissured tongue, tooth-marked tongue, and greasy coating in the male group was higher than that in the female group (*P* < 0.001), whereas the proportion of spotted tongue and stasis tongue in females was significantly higher than that in males (*P* < 0.001). There was no significant difference between the two groups in the proportion of peeled and greasy coating, as shown in Table 4, Figures 7(a) and 7(b).

The results from the above table illustrated that there were significant differences in the incidence of the fissured tongue, tooth-marked tongue, spotted tongue, greasy coating, and rotten coating among the four age gradients, but there was no significant difference in the incidence of stasis tongue and peeled coating. Overall, with the increase of age, the incidence of fissured tongue and greasy coating increased gradually, while the incidence of spotted tongue and tooth-marked tongue decreased gradually, as shown in Table 5, Figures 7(c)–7(d).

### 3.4. Correlation Analysis among Tongue Features and Diseases Based on Complex Networks

**Figure 8:**
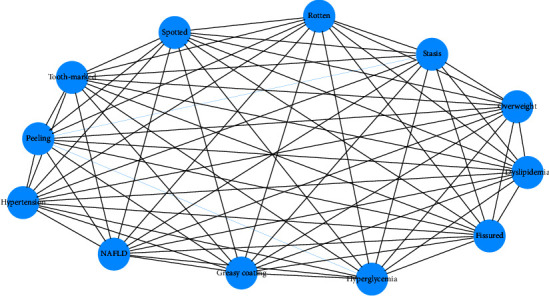
Correlation analysis between tongue features and diseases based on complex network.

Overall, the tongue features of diseases in medical checkups were mainly characterized by increased fissures, tooth marks, and greasy coating.  Table 6 showed the top ten weights relationships between tongue features and diseases. Fissured tongue, tooth-marked tongue, and greasy coating are most closely related to glucolipid metabolic diseases. Specifically, the fissured tongue had the highest weight in hypertension, reaching 0.974, and the weights for dyslipidemia, overweight, and NAFLD were 0.812, 0.799, and 0.775, respectively. For tooth-marked, the weights of hypertension, dyslipidemia overweight and NAFLD were 0.786, 0.649, 0.639, and 0.623, respectively. For greasy coating, the weights of hypertension and overweight were 0.649 and 0.540, respectively. As shown in Figure 8, greasy coating, tooth-marked tongue, and fissured tongue were more closely related to hypertension, dyslipidemia, NAFLD, and overweight.

## 4. Discussion

Intelligent tongue diagnosis is one part of the important content in clinical TCM diagnosis. The researchers have applied the tongue image features extracted by deep learning to diabetes mellitus [4, 42, 43], NAFLD [44], lung cancer-assisted diagnosis [45], and TCM constitution recognition [46–48] with good performance of disease classification [13, 49]. Professor Yang Junlin's team [50] has applied the AI screening system for scoliosis developed and established by Faster R-CNN, and quantified the severity of scoliosis, with the accuracy reaching the average level of human experts. Tang et al. [51] have proposed a tongue image classification model based on multitask CNN, and the classification accuracy achieved 98.33%. However, due to the small sample size, the advantages of deep learning methods cannot be brought into full play, and the tongue features such as rotten, greasy, spotted, stasis, dryness, or thickness remain unexplored [52]. Liu et al. [53] applied Faster R-CNN to identify tooth-marked tongues and fissured tongues, and the accuracy of identifying fissured tongues and tooth-marked tongues was 0.960 and 0.860, respectively. The research only involved tooth marks and fissures due to the small sample size, so the advantages of the deep learning multi-label object detection model were not fully exerted.

Compared with the tongue classification model constructed by the classical CNN, Faster R-CNN as a highly integrated and end-to-end model is still the mainstream object detection neural network at present [54–56].

In our research, we focused on the categories of the tongue image features, rather than the precise feature position, so we applied the method of object detection to the multiclass recognition problem of tongue features. Our tongue feature detection model based on Faster R-CNN had a good generalization ability. With the unique advantages of deep learning and transfer learning in the identification of shape and texture features of tongue images, it can realize automatic high-throughput processing, better solve the problems of local tongue image recognition, integrate the identification and annotation of tongue images, and has a good visualization effect. Our model accomplished the multi-label object detection of 7 categories of tongue images, and the average accuracy achieved 90.67%, showing that Faster R-CNN had a good visualization effect in clinical TCM applications. In addition, the quantitative analysis of tongue features associated with diseases is an important link in the clinical diagnosis of tongue in TCM. The relationship between different features of tongue images, and the association of them with gender/age are not clear [57], and the correlations between them and the occurrence and progress of diseases are unknown. In this study, tongue feature diagnosis based on Faster R-CNN applied to a population undergoing routine medical checkup was a beneficial attempt to mine the implicit information of TCM tongue image and diseases through a complex network [40].

The intelligent diagnostic analysis was established to analyze 3601 physical examination population, and the results showed that the incidence of the fissured tongue was 41.49%, the tooth-marked tongue was 37.16%, the greasy coating was 29.66%, the spotted tongue was 18.66%, stasis tongue was 9.97%, the peeled coating was 3.97%, and the rotten coating was 1.22%, the incidence of fissures, tooth marks and greasy coating in men was higher than that in women, and the incidence of spotted tongue and stasis tongue in women was significantly higher than that in men, which may be related to deficiency of spleen qi, essence and blood in male subjects and excessive blood heat in female subjects. With age increasing, the incidence of fissured tongue and greasy coating increased, while the incidence of spotted tongue and tooth-marked tongue decreased, which may be related to the tendency of both qi and yin deficiency in the elderly and excess syndrome in the young. In the population with glucose and lipid metabolic diseases such as fatty liver and metabolic syndrome, fissures and greasy coating increased, which may be related to the pathogenesis of glucose and lipid metabolism, such as deficiency of qi and yin and dampness. These results were consistent with the clinical practice of TCM [58].

Although the method has some advantages, our model also has limitations.

Firstly, we will conduct further research on the multiclass classification of tongue images in the future. The performance of other neural network models such as VGGnet, ResNet, and DenseNet, will be explored in the task of tongue image classification.

Secondly, the tongue image object detection model has still to be optimized. Annotation of large samples requires a lot of labor cost. Tongue image data acquired by standardized technology has high stability, but the scalability is not strong. Regardless of the fact that the user visualization effect is good, it is still difficult to explain the extracted feature [59]. A more efficient model algorithm, such as unsupervised deep learning based on the flow generation model [60] and a self-attention mechanism based on end-to-end object detection with transformers [61], would be used to further optimize and establish a robust intelligent diagnosis and analysis model of tongue image.

Thirdly, our approach for the detection of tongue images is a qualitative model. However, the identification of tongue images in TCM clinics is complicated, which is not only a binary problem but also a quantification of pathological change. The changes in tongue image features are also of great value in the diagnosis of disease symptoms, which will be the focus of our subsequent research.

## 5. Conclusions

This study was a cross-sectional study of healthy people with medical checkups. Furthermore, a case-control study will be carried out on patients with major chronic diseases in order to prove the value of tongue features in the diagnosis of disease. In addition, we will optimize the Faster R-CNN model with the respect to the precise location of objects in a tongue image. This paper presents a supervised deep learning method based on a large amount of labeled data. In the future, we will explore a more robust self-supervised deep learning model for the multiclassification of tongue features.

The model Faster R-CNN shows good performance in tongue image classification. And we have preliminarily revealed the relationship between tongue features and gender, age, and metabolic diseases in a medical checkup population.

## Figures and Tables

**Table 1:** The training set.

Tongue image categories	Training datasets	Labels
Fissured tongue	1570	1792
Tooth-marked tongue	1386	2589
Spotted tongue	746	920
Stasis tongue	1107	1942
Greasy coating	1559	1652
Peeled coating	478	639
Rotten coating	96	132
Total	6942	9666

Notes: one tongue image can contain multiple labels.

**Table 2:** Initial parameters of faster R-CNN model for training.

Parameters	Values
Base learning rate	0.03
Weight decay	0.0001
Momentum	0.9
Gamma value	0.1
Steps	(0, 13333, 26666)
Max iteration	40000
Scales	400, 500
Batch size	128

**Table 3:** Tongue images object detection results based on Faster R-CNN.

Tongue feature	Precision (%)	Recall (%)	F1-score (%)	Accuracy (%)
Fissured	99.49	99.49	99.49	98.97
Tooth-marked	100.00	98.84	99.42	98.84
Stasis	99.22	93.43	96.23	92.75
Spot	98.73	84.78	91.22	83.87
Greasy	99.44	90.72	94.88	90.26
Peel	98.11	88.14	92.86	86.67
Rot	100.00	83.33	90.91	83.33
Average	99.28	91.25	95.00	90.67

**Table 4:** Comparison of tongue image features between different genders.

	Male (*n* = 2006)	Female (*n* = 1595)	*χ* ^2^	*P*
Fissure (yes)	978 (48.8%)	516 (32.4%)	98.475	<0.001
Tooth (yes)	794 (39.6%)	544 (34.1%)	11.405	<0.001
Spot (yes)	336 (16.7%)	336 (21.1%)	10.904	0.001
Stasis (yes)	92 (4.6%)	267 (16.7%)	146.223	<0.001
Greasy (yes)	569 (53.3%)	499 (46.7%)	3.632	0.057
Peel (yes)	84 (4.2%)	59 (3.7%)	0.556	0.456
Rot (yes)	37 (1.8%)	7 (0.4%)	14.544	<0.001

**Table 5:** Comparison of tongue image features among different age ranges.

	<30 years (*n* = 848)	30–39 years (*n* = 1418)	40–49 years (*n* = 826)	≥50 years (*n* = 509)
Fissure (yes)	299 (35.3%)	510 (36.0%)	382 (46.2%)^*∗*^^#^	303 (59.5%)^*∗*^^#▲^
Tooth (yes)	321 (37.9%)	580 (40.9%)	302 (36.6%)	135 (26.5%)^*∗*^^#▲^
Spot (yes)	249 (29.4%)	291 (20.5%)^*∗*^	94 (11.4%)^*∗*^^#^	38 (7.5%)^*∗*^^#^
Stasis (yes)	81 (9.6%)	150 (10.6%)	92 (11.1%)	36 (7.1%)
Greasy (yes)	205 (24.2%)	391 (27.6%)	273 (33.1%)^*∗*^^#^	199 (39.1%)^*∗*^^#^
Peel (yes)	27 (3.2%)	60 (4.2%)	33 (4.0%)	23 (4.5%)
Rot (yes)	3 (0.4%)	13 (0.9%)	8 (1.0%)	20 (3.9%)^*∗*^^#▲^

Note: ^*∗*^ denotes significant difference compared to < 3 0 years old group, # denotes significant difference compared to 30–39 years old group, and ▲ denotes significant difference compared to 40–49 years old group.

**Table 6:** Top 10 weight of tongue features and diseases in medical checkups.

Tongue feature	Disease	Weight
Fissured tongue	Hypertension	0.974
	Dyslipidemia	0.812
	Overweight	0.799
	NAFLD	0.775

Tooth-marked tongue	Hypertension	0.786
	Dyslipidemia	0.649
	Overweight	0.639
	NAFLD	0.623

Greasy coating	Hypertension	0.649
	Overweight	0.540

## Data Availability

The datasets used and/or analyzed in this study are available upon reasonable request from the corresponding author.
